# The gerontological nurse specialist’s core competencies in China: A cross‐sectional study

**DOI:** 10.1002/nop2.583

**Published:** 2020-09-28

**Authors:** Bai Chunlan, Pu Lihui, Chen Hongxiu, Hu Xiuying

**Affiliations:** ^1^ West China School of Nursing Sichuan University Chengdu China; ^2^ Griffith University Queensland Australia; ^3^ Innovative Nursing Research Center School Of Medicine West China Hospital Sichuan University West China Hospital of Sichuan University Chengdu China

**Keywords:** core competencies, geriatric nursing, gerontological nurse specialists

## Abstract

**Aim:**

**To** describe the core competencies of gerontological nurse specialists and investigate the factors that contribute to the development of core competencies.

**Design:**

A descriptive cross‐sectional study was conducted from August 2015–March 2016.

**Methods:**

The descriptive study of gerontological nurse specialists’ core competencies used a self‐assessment instrument with three first‐level domains (attitude, skill and knowledge) and 9 s‐level dimensions. A total of 225 gerontological nurse specialists from ten provinces in China were selected by a convenience sampling method.

**Results:**

The average core competency score of the gerontological nurse specialists was 3.78, in the middle level(3.30–4.17/5.00). Core competency was associated with gerontological nurse specialists’ age (*p* = .005), professional title (*p* = .017), hospital level (*p* = .006) and passion for geriatric nursing (*p* = .000). The average scores of attitude, skill and knowledge were 4.45, 4.02 and 4.18, respectively. All first‐level domains were related to age (*p* ≤ .021) and passion for geriatric nursing (*p* ≤ .008); knowledge and skill were associated with professional title (*p* ≤ .045) and attitude and skill were associated with hospital level (*p* ≤ .020).

## 1 INTRODUCTION

At the end of 2018, there were 249 million people aged 65 years or over in China, accounting for 11.4% of the total population (National Bureau of Statistics, 2018). In China, 74.2% of adults older than 60 years have at least 1 common chronic disease (Cui, Mao, & Wang, 2016). Due to the ageing of their bodily organs, it is difficult for them to maintain their physical and psychological balance (Fried, Ferrucci, Darer, Williamson, & Anderson, 2004). A national survey indicated that 59.2% of older inpatients were over 80 years old, the average number of hospitalizations tripled with increasing age and the number of re‐hospitalizations increased significantly (Xu, et al, 2014). Older adults experience greater health risks and have a particularly increased demand for hospital care (Mara, Freitas, Flavia, & Cristina, 2017).

Gerontological nurse specialists (GNSs) engage more in the application of elderly care best practices (Conley et al., 2012; Cora, & Lee, 2016) than Advanced Practice Registered Nurses (APRNs) and Registered Nurses (RNs) when assessing the condition and taking care of older adults in hospitals (Dodge, 2010). Previous studies demonstrated that GNSs can effectively meet the health needs of older adults and improve their outcomes (Jiménez, María, Gómara, & Germán, Concha, 2015). Thus, in 2012, the Sichuan Provincial Nurses Association, under the leadership of the Chinese Nursing Association, established GNS training centres and began programmes to train GNSs. Subsequently, other nursing associations also gradually began to cultivate GNSs in China. The training curricula are designed by associations. The length of the geriatric specialized theoretical courses varies from 1–3 months and the length of the geriatric specialized clinical practice courses varies from 1–12 months. There are no standard GNS training programmes. Until 2016, it was preliminarily estimated that approximately 400 nurses throughout the country had completed the training in Sichuan GNS training centres.

## 2 BACKGROUND

The core competencies were used as the standard for nursing administrators to evaluate nurses and determine their strengths and the areas in need of improvement (Conley, 2011), and they were the key to ensuring the provision of high‐quality nursing services (Cutugno, 2015). Many studies on the competency of GNSs have been performed. These studies have identified the role of GNSs through qualitative methods (Jonsson, Hofoss, Kirkevold, & Foss, 2016), constructed a competence index (Cui & Feng, 2019; Huizenga, Finnema, & Roodbol, 2016; Liu et al., 2016), tested the reliability and validity of a self‐made competence questionnaire (Kilpatrick et al., 2013) and assessed the current status of GNSs’ emotional competence (Bahrami, Purfarzad, Keshvari, Rafiei, & Sivertsen, 2018). The subjects were recruited from long‐term institutions or nursing homes (Huizenga et al., 2016; Jonsson et al., 2016) rather than from hospitals. A recent study in Israel described the professional competencies of gerontological nurse practitioners and found that they had a positive attitude about their clinical role but that their professional knowledge and skills could be improved (Yafa, Dorit, & Shoshana, 2016). However, few studies have described the core competencies of GNSs (Boscart, Mccleary, Huson, Sheiban, & Harvey, 2016), especially those working in hospitals.

However, 60% of medical–surgical patients were older adults and 46% were critical care patients, accounting for 50% of the patients in intensive care units in hospitals (Mezey, Stierle, Huba, & Esterson, 2007). Thus, the core competencies of GNSs in hospitals have a large impact on the health outcomes of older adults. In addition, the core competencies of GNSs also have an impact on the clinical skills and professional identity of nursing students (Hou, Zhu, & Zheng, 2011). Additionally, studies on the core competencies of GNSs also indicate the significance of clinical research, education and the career development of nurses in various countries (Chen & Wang, 2016). Therefore, this study describes the core competencies of GNSs working in Chinese hospitals from their own perspective, focusing on their attitude, skill and knowledge and investigated factors that can contribute to the development of core competencies.

## 3 METHOD

### 3.1 Design

This was a cross‐sectional, descriptive study involving a self‐assessment questionnaire. Convenience sampling was used to recruit participants who had completed the GNS training course of the Sichuan Provincial Nurses Association and still worked at the hospital. The GNS training was implemented at West China Hospital of Sichuan University and Sichuan Provincial Hospital. From August 2015–March 2016, we contacted GNSs (*N* = 320) and the response rate was 79.7% (*N* = 255). Questionnaires with the same answer for every question or with more than 3 missing answers were excluded. Finally, 225 valid questionnaires were collected. One hundred eighty‐one of the GNSs came from Sichuan Province and another 44 were from nine different provinces in China. Over half of them received the GNS certificate at West China Hospital of Sichuan University.

### 3.2 Instrument

The “Gerontological nurse specialists’ core competencies assessment questionnaire” collected the basic demographic information and the competence characteristics of the GNSs. The questionnaire included three first‐level domains (Attitude, Skill, Knowledge), nine second‐level dimensions (Professional self‐identity, Learning enthusiasm, Clinical nursing skill, Communication management and research skills, Analysis/judgment decision‐making skills, Legal/ethical practice skills, Professional development skills, Basic knowledge, Professional knowledge) and 69 third‐level items reflecting current the knowledge base and practice scope of the GNSs. The Cronbach's ɑ of the overall questionnaire was 0.98 and the Cronbach's α of the second‐level dimensions ranged from 0.83–0.96 (Chen et al., 2019).

The questionnaire used a 5‐point response scale for the GNS to indicate the extent to which they agreed or disagreed with each core competency item. The response options were strongly agree, partly agree, agree, partly disagree and strongly disagree. Each core competency was categorized as low (<3.50), moderate (3.50–4.49) or high (≥4.50) based on the self‐assessment score.

### 3.3 Data collection

With the help of the Sichuan Provincial Nurses Association and the two training hospitals, we obtained the name, email addresses and hospital workplace of the GNSs. Then, two researchers on the team distributed questionnaires by hand to those who worked in Chengdu, the capital city of Sichuan Province and collected questionnaires on the same day. If a GNS worked in another city, we sent emails and a reminder email was sent to those who did not respond within two weeks. Ultimately, 79 questionnaires were collected by email.

### 3.4 Data analysis

The basic demographic information (age, gender and education) and the professional characteristics of the GNSs (professional title, hospital level, working department and interest in older adult care) were described as percentages based on the number of responses. Medians and quartile ranges were used to describe the 69 core competencies of the GNSs, as the score of the core competencies was abnormally distributed. The relationships among the demographic information, professional characteristics and core competencies were examined by the Mann–Whitney U test or Kruskal–Wallis H test.

### 3.5 Ethical consideration

This study was reviewed and orally approved by the ethics committee of West China Hospital of Sichuan University. Before the study, we distributed a letter of invitation and the questionnaire to GNSs to explain the study's purpose and the voluntary nature of participation. The GNSs were also informed that the return of the questionnaire would be interpreted as their consent to participate.

## 4 RESULTS

### 4.1 Participants’ characteristics

The study included 225 GNSs, 98.2% of whom were female, with a mean age of 29 years (lower quartile, 26; upper quartile, 33). In total, 61.8% of them were senior nurses and 83.5% worked at level three class A hospitals. Almost 80.0% of the GNSs were interested in their job. Table 1 presents details of the basic demographic information and the professional characteristics of the GNSs.

**Table 1:** Gerontological nurse specialists’ demographic information (*N* = 225)

Variable	Total		Variable	Total	
	*n*	(%)		*n*	(%)
**Age** (years)			**Hospital level**		
20–29	90	40.0	Level three class A	167	74.2
30–39	114	50.7	Level three class B	21	9.3
≥40	21	9.3	Level two class A	27	12.0
**Sex**			Level two class B	4	1.8
Male	4	1.8	Other	6	2.7
Female	221	98.2	**Department**		
**Education**			Geriatrics	153	68.0
Diploma degree	68	30.2	Other	72	32.0
Bachelor's degree or above	157	69.8	**Interest in geriatric nursing**		
**Professional title**			Like very much	48	21.3
Nurse	16	7.1	Like	132	58.7
Senior nurse	139	61.8	Normal	45	20.0
Nurse‐in‐charge	62	27.6	Dislike	0	0.0
Professor of nursing	8	3.5			

Note

Senior nurse: conduct clinical nursing work and technical operations and teach nursing students.

Nurse‐in‐charge: intermediate professional title, inspect and supervise the clinical work of nurses.

Professor of nursing: senior professional title, manage clinical staff and promote professional development.

Level two class B hospital: provides health services across several communities.

Level two class A hospital: provides health services across several communities and departments, medical staff, hospital management, medical technical and facilities are better than the level two class B hospital.

Level three class B hospital: provide medical and health services across regions, provinces and cities as well as across the country.

Level three class A hospital: The highest level hospital in China, which provides medical and health services across regions, provinces and cities as well as across the country and departments, medical staff, hospital management, medical techniques and facilities are better.

Geriatrics department: All patients are older adults. It can take care of all diseases of older adults and provide comprehensive treatment.

Interest in geriatric nursing: passion for geriatric nursing and taking responsibility for their work.

### 4.2 The core competencies of the GNSs

The GNSs evaluated their core competencies as moderate (3.78) and the score for attitude (4.45) was better than that for knowledge (4.18) and skill (4.02).

#### 4.2.1 Attitude

The attitude of professional self‐identity was high (4.67). GNSs understand the care needs of older adults and treat each person fairly and they do anything they can to promote the improvement of patients’ quality of life. The GNSs love and are proud of their job and they are willing to influence the development of geriatric nursing. The other attitude was learning enthusiasm, which was moderate (4.20). The GNSs lack enthusiasm for actively acquiring advanced knowledge and cooperating with relevant departments to provide personalized care and improve care quality.

#### 4.2.2 Skill

Legal/ethical practice skills (4.75) were rated the highest in terms of competency. The GNSs know how to follow the law and regulations to protect themselves from lawsuits and obey the ethical principles to keep inpatients’ conditions confidential and maintain their families’ privacy. In contrast, GNSs’ lowest‐rated skill (3.75) was professional development. GNSs lack awareness and methods to proactively obtain literature and advanced information about geriatric nursing, both nationally and internationally and they do not have insight into their professional strengths and weaknesses; thus, they cannot make career development plans to match their professional characteristics. Furthermore, both the clinical nursing skills (4.20) and the analysis/judgement decision‐making skills (4.20) of the GNSs were moderate. The GNSs lacked the ability to assess older adults’ health problems and drug management problems, make care plans according to such an assessment, provide personalized medical care and help older adults understand the risks, benefits and outcomes of different treatments. Another middle‐level competence was communication management and research skills (3.77). The GNSs reported that they lack opportunity, time and ability to communicate with older inpatients and their caregivers, implement evidence‐based practice and conduct research during clinical work.

#### 4.2.3 Knowledge

The GNSs reported that they had middle‐level knowledge (4.18) and their professional geriatric nursing knowledge (4.29), such as ageing, comprehensive syndromes of older adults, common chronic diseases, safety prevention, end‐of‐life care, professional nursing skills and community home care, was greater than their basic nursing knowledge (4.00), such as nursing ethics, health promotion, disease prevention and nursing research.

### 4.3 Description and comparisons of GNSs’ core competencies and characteristics

When the core competency scores were analysed according to the GNSs’ independent variables of age, professional title, hospital level, working department and interest in geriatric nursing, statistically significant (*p* ≤ .05) differences were found except for the working department (*p* = .081). GNSs who were 40 years and older had statistically higher competency levels *(p* = .005), regardless of attitude, skill and knowledge, than those between 20–39 years old. GNS professors had the highest geriatric nursing competency levels (*p* = .017), both in skill and knowledge. The attitude towards geriatric nursing did not differ by professional title (*p* = .088). GNSs working at level three class A hospitals had higher competency levels (*p* = .006) in terms of attitude and skill than other GNSs. In clinical work, GNSs who liked their job very much had the highest core competency levels (*p* = .000) in attitude, skill and knowledge.

## 5 DISCUSSION

This study presents important results on the core competencies and influencing factors of GNSs in China. Currently, GNSs’ attitude is better than their skill and knowledge and the core competencies are moderate, especially communication skills, research skills and professional development skills. The core competencies were related to age, professional title, hospital level and passion for geriatric nursing.

Currently, GNSs had a positive attitude towards elderly care in China. This result corroborates previous study results (Yafa et al., 2016) and may be explained by the fact that professional training and clinical practice benefited their professional identity (Haron, Levy, Albagli, & Riba, 2013). Furthermore, many studies agree that interest in older adult care affects core competency (Li, Li, Chen, Li, & Xiao, 2017; Yang, Zhou, & Cheng, 2017), perhaps because GNSs’ passion for their work can improve their job satisfaction (Xu, Fu, 2019; Wu et al., 2018), motivate them towards self‐improvement of skills and knowledge and make them more passionate and enthusiastic in providing more efficient care services for older adults.

Although the passion of elderly nursing, professional training and clinical experience can improve skills (Esterson, Bazile, Mezey, Cortes, & &Huba, 2013), the skill of GNSs in China was at a medium level, especially communication skills, research skills and professional development skills. The reason for GNSs’ poor communication skills may be that GNSs cannot provide useful professional information and satisfactory care services to establish an effective and good communication relationship with family caregivers (Louise, Bourbonnais, Bernier, & Benoit, 2017; Liesbeth, Vliet, Elizabeth, Julia, & Weert, 2015). Additionally, with their heavy clinical workload, there is little time for GNSs to communicate with older adults and their family caregivers (Beth, Ellen, Barba, Hu, & Efird, 2012; Li et al., 2017). Their scientific research and professional development skills were insufficient, similar to those of other types of nurses in China (Cao, Wang, Liang, & Chen, 2018; Li et al., 2017; Qi & &Wang, 2016). This may be because GNSs are mainly focused on direct patient care in clinical work like other nurses, so they have limited awareness of scientific research and the development of professional skills. Additionally, the skills of GNSs were not related to work department in this study, confirming the result of a previous study (Rawson, Bennett, & Ockerby, 2017). However, Wei, Niu and Ge described that the department had an impact on the core competencies of ICU nurses (2018). The reason may be that GNSs work not only in the geriatric department but also in other departments and with the growth of the older adult population, most departments have a large number of older adults in China. Furthermore, there is no clear professional identification of GNSs in China; GNSs are registered as nurses in the clinical environment, so they may not focus on geriatric nursing, conduct research or think about professional development (Huizenga et al., 2016; Kilpatrick et al., 2013; Wangensteen, Finnbakk, & Adolfsson, 2018). Considering all the reasons above, GNSs should cooperate as a multi‐disciplinary team member to communicate with family members about medical information and as a researcher on the team. Nursing managers need to clearly define the responsibilities of GNSs as they do for other specialized nurses in the clinical work climate (Purfarzad et al., 2019), make GNSs contribute to ward management, provide patient‐centred care (Fukada, 2018) and have sufficient time and opportunity to focus on geriatric nursing and improve their professional skills.

GNSs’ basic knowledge was better than their professional knowledge, and there was no difference in knowledge between GNSs working at different levels of hospitals. In China, older adults are more likely to choose high‐level hospitals when they are sick (Shao, Tian, & Li, 2012); approximately half of the inpatients in high‐level hospitals are older adults and the proportion is rising (Xu, Ma, Luo, & Wang, 2013). Therefore, GNSs at these hospitals have more opportunities to understand diseases of ageing (Rawson, 2017; Henni, Kirkevold, Antypas, & Foss, 2018; Koroknay, 2015). The results demonstrated that GNSs mainly focused on direct patient care (Huizenga et al., 2016) and that there was no professional role of GNSs in clinical work, such that they had limited awareness of the complete range of knowledge as a health advocate, scholar and professional. Meanwhile, GNSs lack the means and resources to obtain international and advanced professional information in clinical working environments (Huizenga et al., 2016). To improve professional knowledge, nursing managers need to not only use human resources appropriately but also provide more opportunities for continuing education(Shaheen, El‐Hneiti, Albqoor, & Ahmad, 2019), which could provide the resources for GNSs to gain advanced and professional knowledge (Ann, Huang, Liu, Fans, & Yao, 2018). Regular and planned refresher training also had a positive impact on clinical practice (Bahceci, Mine, Celebioglu, Ayda, 2017).

Overall, the GNS training programme does not adequately improve professional skills and knowledge in clinical work in China. One of the reason may be that the cultivation and training of geriatric nurses are still developing in China. In 2010, Tianjin University of Traditional Chinese Medicine began an undergraduate education programme in geriatric nursing (xu, Fu, 2019) and the GNS training programme began in 2012, which varied between different training centres until now. In the future, a standard GNS training programme should be used to ensure that nurses can obtain professional skills and knowledge. Another reason may be that the entry education level of GNSs is a bachelor's degree in other countries(American Nurses Credentialing Center; Canadian Nurses Association); however, only 30% of GNSs have a diploma degree in China, so trained nurses may not fully understand the contents of the training. The educational background of the nurses (Selvig, Holaday, Purkiss, Hortsch, 2015) should be considered in future training. In this study, the core competencies of GNSs were related to age and professional title. Older nurses may understand patients and their care needs more deeply (Raquel, Guillermo, Montoya, García, & Hueso, 2019). A higher professional title indicates more professional training or more clinical experience, which is very important for improving nursing skills and knowledge (Esterson et al., 2013). Nursing managers can provide continuing education according to the age and professional title and the older GNSs and those with a higher professional title can be team leaders to share their clinical experience with other GNSs to promote core competence.

### 5.1 Limitations

In this study, the convenience sampling of subjects limits the generalization of our results. Therefore, future studies are needed to validate the core competency scale with a larger sample size. In addition, the self‐assessment may show falsely high competencies.

### 5.2 Implications for nursing policy and practice

The findings can be used as a foundation to develop questionnaires to assess the core competencies of GNSs. The results of GNSs’ core competence contribute to the GNS training course and nursing managers and nursing school teachers should adjust the training content and method and improve core competence according to the characteristics of GNSs. GNSs can also use the findings for self‐cultivation in clinical practice or as motivation to participate in continuing education and other professional training to development their competency. Future research should explore interventions to improve the professional skills and knowledge of GNSs and verify whether they are effective by the assessment questionnaire.

## 6 CONCLUSION

GNSs play a very important role in geriatric nursing. This study showed that GNSs reported moderate levels of core competency and their communication skills, research skills and professional development skills need to be further developed. The core competency training and the clinical practice of GNSs should be taken into account to improve GNSs’ competency in the future, according to GNSs’ personal characteristics.

## CONFLICT OF INTEREST

The authors declare no conflict of interest.

## AUTHOR CONTRIBUTIONS

All authors: study conception/design; Bai, Pu and Chen: data collection/analysis; Bai: manuscript drafting; Bai and Pu: critical revisions for important intellectual content/supervision/statistical expertise; Pu and Hu: administrative/technical/material support; All author: revision and final approval of manuscript.

7

**Table 2:** Gerontological nurse specialists’ core competency scores

	P_50_	**P_25_‐ P _75_**
**Attitude**	**4.45**	**3.82–4.82**
A−1‐ Professional self‐identity	4.67	4.00–5.00
A−2‐ Learning enthusiasm	4.20	3.60–4.80
**Skill**	**4.02**	**3.45–4.67**
S−1‐ Clinical nursing skills	4.20	3.53–4.67
S−2‐ Communication management and research skills	3.77	3.08–4.38
S−3‐ Analysis/judgment decision‐making skills	4.20	3.50–4.80
S−4‐ Legal/ethical practice skills	4.75	4.00–5.00
S−5‐ Professional development skills	3.75	3.00–4.25
**Knowledge**	**4.18**	**3.53–4.70**
K−1‐ Basic knowledge	4.00	3.33–4.67
k−2‐ Professional knowledge	4.29	3.61–4.75
**Overall score**	**3.78**	**3.30–4.17**

Note

Competency score ranges from 1 (strongly disagree)–5 (strongly agree).

**Table 3:** The core competencies of gerontological nurse specialists with different characteristics

Variable	Attitude	Skill	Knowledge	Total
**Age (years)**				
20–29 (*N* = 90)	108.92	110.95	110.02	110.13
30–39 (*N* = 114)	109.32	106.82	108.16	107.21
≥40 (*N* = 21)	150.45	155.33	152.02	156.71
**P value**	0.021*	0.007^**^	0.015*	0.005^**^
**Professional title**				
Nurse (*N* = 60)	123.63	142.25	114.56	136.56
Senior nurse (*N* = 139)	128.17	102.95	104.45	102.89
Nurse‐in‐charge (*N* = 62)	104.36	122.18	126.73	124.65
Professor of nursing (*N* = 8)	124.00	158.00	151.94	151.31
**P value**	0.088	0.009^**^	0.045*	0.017*
**Hospital level**				
Level three class A (*N* = 167)	118.92	100.89	115.48	120.10
Other (*N* = 58)	95.95	90.70	105.85	92.56
**P value**	0.020*	0.002^**^	0.331	0.006^**^
**Department**				
Geriatrics (*N* = 153)	115.63	118.70	114.52	118.20
Other (*N* = 72)	107.42	100.89	109.76	101.94
**P value**	0.376	0.056	0.573	0.081
**Interest in geriatric nursing**				
Like very much (*N* = 48)	155.78	140.63	138.64	147.34
Like (*N* = 132)	105.60	105.56	105.26	105.47
Normal (*N* = 45)	88.98	105.34	108.36	98.46
**P value**	0.000^**^	0.004^**^	0.008^**^	0.000^**^

Note

Mann–Whitney U test, average rank to describe the core competencies

* is significantly different when p﹤0.05; ^**^is significantly different when p﹤0.01.
